# Need for Mechanisms to Monitor Ocean Circulation‐Driven Seagrass Population Expansions

**DOI:** 10.1002/ece3.71087

**Published:** 2025-03-27

**Authors:** Zhaohua Wang, Bin Zhou, Wenjie Yan

**Affiliations:** ^1^ First Institute of Oceanography Ministry of Natural Resources Qingdao China; ^2^ Observation and Research Station of Bohai Strait Eco‐Corridor First Institute of Oceanography, Ministry of Natural Resources Qingdao China; ^3^ College of Marine Life Science, Ocean University of China Qingdao China; ^4^ Key Laboratory of Mariculture (Ocean University of China), Ministry of Education Qingdao China; ^5^ Key Laboratory of Ecological Prewarning, Protection and Restoration of Bohai Sea, Ministry of Natural Resources Qingdao China; ^6^ Observation and Research Station of Yellow‐Bohai Sea Temperate Seagrass Bed Ecosystem, Ministry of Natural Resources Qingdao China

**Keywords:** colonization pathway, dispersal, drive, evolution, genetic structure, *Zostera marina*

## Abstract

Seagrass beds are increasingly degraded; however, their protection and restoration are still confined to localized marine areas, making it difficult to establish connectivity between differently protected and restored areas. One limiting factor is the lack of understanding of the processes and mechanisms contributing to seagrass population expansion at the ocean area scale, which is the main driver of seagrass dispersal via oceanic circulation. Coastal China. Taxon: Eelgrass ( *Zostera marina* )Using eelgrass as a model species, we propose a strategy to resequence seagrass samples from different geographical populations, analyze the genetic structure of seagrasses by combining genomics and population evolution, construct and screen an optimal model of seagrass colonization history, calibrate the timing of colonization events, and thereby deduce the evolutionary history of seagrass populations. We constructed a three‐dimensional hydrodynamic model based on the FVCOM numerical model to clarify the seasonal changes in the surface circulation patterns of seagrasses in their natural distribution areas and to precisely locate the colonization pathways of seagrass populations by combining the history of population differentiation. This study elucidated the main proliferation pathways of the target seagrass populations and their physical driving mechanisms and provides a theoretical basis for the study of seagrass population evolution and their protection and restoration.

## Graphical abstract

The degradation of seagrass beds along the coast of China is worsening. Their protection and restoration are confined to local marine areas, making it difficult to establish connectivity between different regions. One of the limiting factors is the lack of understanding of the mechanisms of seagrass population expansion. Using eelgrass ( *Zostera marina* ) as a model species, we deduced the evolutionary history of seagrass populations through methods such as resequencing seagrass samples, analyzing the genetic structure, and constructing and screening models. Based on the FVCOM numerical model, a three‐dimensional hydrodynamic model was constructed to clarify the seasonal changes in the surface circulation patterns of seagrasses in their natural distribution areas. By combining the history of population differentiation, the colonization pathways of seagrass populations were precisely located. This study provides a theoretical basis for the study of seagrass population evolution as well as their protection and restoration.

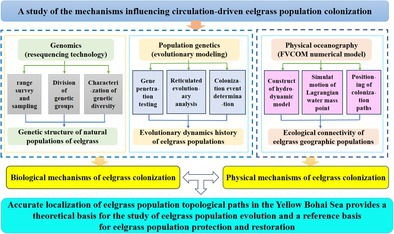

## 1 Introduction

Seagrasses are the only group of angiosperms that have returned to the ocean from terrestrial environments and live exclusively in seawater (Fahimipour et al. 2017), with more than 70 known constituent species (Huang et al. 2018), distributed over thousands of kilometers of sedimentary shoreline from the subarctic to the tropics (Duarte and Krause‐Jensen 2017), where they occur in shallow marine ecosystems in coastal areas (Yu et al. 2023). Large, continuously distributed seagrass beds consisting of one or more seagrass species act as “forests in the sea” and are rich in ecological functions and biodiversity, providing high primary productivity, organic carbon sequestration and accumulation, and sediment stabilization (Douglas 2021). They provide habitats and food for marine fish and invertebrates, and serve as important spawning and nursery grounds (Heckwolf et al. 2021). Seagrass beds have thus become typical offshore marine ecosystems worldwide (Reusch et al. 2021).

Seagrass beds worldwide have become highly vulnerable and degraded because of global climate change and anthropogenic impacts (Orth et al. 2006; Olsen et al. 2016; Reusch et al. 2021). Globally, seagrasses are declining at an alarming rate (~110 km^2^ per year), and 29% of the global seagrass population has died out by the end of 2006. For example, only in the nearshore areas of Shandong, China, the distribution of seagrass beds has decreased by more than 80% (Huang et al. 2015). Among seagrasses, eelgrass ( *Zostera marina* ) is the species with the widest global geographical distribution and is a typical Northern Hemisphere temperate seagrass species that is distributed in both the Pacific and Atlantic regions from warm temperate to icy Arctic environments, spanning latitudes of 40° (Green and Short 2003). Seagrass beds in China are divided into two main distribution areas, namely the South China Sea and Yellow Bohai Sea seagrass areas. The latter includes the coasts of the Shandong, Hebei, Tianjin, and Liaoning provinces (Zhou et al. 2023).

Aside from having the largest distribution area, eelgrass is the seagrass species with the highest biomass and is the dominant species in the Yellow Bohai Sea seagrass distribution area (Zhou et al. 2023). In the following section, we consider eelgrass as a model organism on which this study is based. It has been shown that the geographical distribution of eelgrass in China has shifted northward during recent decades (Xu et al. 2022), and the eelgrass beds originally distributed in Rizhao, Shandong Province, have disappeared. Concurrently, the southern boundary of the distribution of eelgrass in China has shifted northward to the vicinity of Qingdao Yumingzui. Eelgrass beds in China face persistent threats from both natural and anthropogenic factors, and there are no other seagrass species able to fill the vacant ecological niches created by the degradation of eelgrass beds (Green and Short 2003). Therefore, there is an urgent need to conserve and restore eelgrass beds in China and improve their ecological functions. However, the conservation and restoration of eelgrass beds have been largely based on localized efforts, and there is a lack of analysis of the connectivity between different marine protected areas and restoration zones, assessment of the feasibility of germplasm exchange and utilization of different eelgrass geographical populations, and design of multi‐location synergistic conservation and restoration strategies based on the natural dispersal of seagrass populations. A fundamental limitation is the lack of understanding of the processes and mechanisms underlying eelgrass population expansion at the regional scale.

A study of the expansion process and mechanisms affecting eelgrass populations at a regional scale requires the integration of several disciplines, such as seagrass population genetics and physical oceanography, and focuses on the application of population genetics and biogeography based on the biological and genetic characteristics of seagrasses. This needs to be considered in combination with an analysis of regional oceanic circulation patterns, to determine the ecological circulation process between different geographical seagrass populations and to identify their origins and expansion pathways. Many field surveys have shown that the phenotypic characteristics and developmental status of different seagrass geographic populations are significantly different, indicating that seagrasses have differentiated into different genetic groups during the evolutionary process. Species formation and differentiation is a continuous process (Kern and Hahn 2018) and the molecular mechanisms underlying this can be elucidated by studying the differentiation process and genetic structure of the target group (Wang et al. 2019). With the continuous innovation in sequencing technology, there has been a massive increase in studies revealing genetic variation in the evolutionary process of different biological species using next‐generation sequencing (Yan, Wang, and Zhou 2023), especially in terrestrial higher plants and marine organisms (e.g., fish and shellfish). Therefore, resequencing technology can be applied to combine phylogenetic genomics and population genetics theoretical approaches to systematically study the phylogenetic relationships and differentiation history among natural populations of seagrasses in the target area (Yan, Wang, and Zhou 2023).

Higher plants reproduce and disperse seeds in several ways. As a higher marine plant, eelgrass has evolved a unique hybrid reproductive system to adapt to the marine environment (Xu et al. 2020), which includes both asexual reproduction based on stolon growth and sexual reproduction based on seeds. Seed dispersal allows eelgrasses to expand their territories, reproduce, and increase their ability to adapt to different environmental conditions (Xu et al. 2020). In particular, the exchange of genes among different geographical populations and population expansions is mainly dependent on the widespread dispersal of seeds. As such, dispersal is a physical factor that plays a decisive role (van Gennip et al. 2017).

As an angiosperm living entirely in seawater (Gamble et al. 2021), eelgrass reproductive branches or seeds can reach different ecological patches through seawater transport processes such as oceanic circulation. Eelgrass reproductive branches with seeds can survive for several weeks (Gamble et al. 2021), which theoretically allows long‐distance dispersal using ocean currents. Therefore, by relying on ocean circulation movements, eelgrass can realize long‐distance seed dispersal, increasing the chances of producing offspring and promoting genetic exchange among different geographical populations, thus greatly expanding the distribution range of the species and improving ecological connectivity among patches (Wu et al. 2018, Wu, Milne, et al. 2023). Ecological connectivity is an important indicator of ecosystem integrity (Siebenhüner 2007; Du et al. 2015), and maintaining connectivity among habitat patches is key to enhancing population size and structure, as well as the genetic diversity of species among habitats and maintaining biodiversity (Williamson et al. 2016). Therefore, studying the processes and mechanisms of ocean circulation‐driven eelgrass reproductive branching or seed dispersal over long distances is important for guiding the conservation and restoration of eelgrass beds (Zhao et al. 2023).

Ocean circulation is a unique driver of coastal geographic processes (van Gennip et al. 2017; Lin et al. 2019). In this study, we focused on ocean circulation motion at the sea area scale. This regional circulation pattern has obvious seasonality, with its direction and intensity showing large differences between the different seasons (Wu, Zhai, et al. 2023). Circulation can be further divided into boundary, surface, middle, and bottom layers (Wu, Zhai, et al. 2023), which correspond to the different levels of life of different species and require different levels of circulation. Seagrasses grow mostly in shallow subtidal to intertidal areas. Therefore, focusing on the seasonal characteristics of regional surface currents could elucidate the physical driving mechanism of oceanic circulation that promotes long‐distance dispersal of eelgrass reproductive shoots (or seeds) to achieve colonization of different habitats.

As an important component of ocean systems, there is a close link between oceanic circulation and marine ecosystems. For example, circulation plays an important role in regulating the distribution of and changes in seawater temperature, salinity, and nutrient content. These factors directly and indirectly affect the survival and reproduction of marine organisms. Numerous studies have shown that temperature plays a decisive role in eelgrass growth (Yan et al. 2023a, 2023b). Especially, abnormally high water temperatures may cause devastating damage to eelgrass seedlings (Wang et al. 2023). In summary, an in‐depth study of the mechanisms of eelgrass population dispersal and evolution driven by oceanic circulation is crucial for elucidating the mechanisms that maintain ecological connectivity between different geographical populations (Wu et al. 2018, Wu, Milne, et al. 2023).

Global degradation of seagrass beds is becoming increasingly serious. Relevant conservation and restoration studies and initiatives are still limited to localized areas, making it difficult to establish ecological connectivity among different distribution areas (Lu et al. 2023). One of the main constraints affecting this situation is the lack of understanding of the regional processes and mechanisms of the seagrass population. Statistically, studies on seagrass population conservation and ecological connectivity have focused on biological, geological, and chemical processes and human impacts, whereas few studies have focused on the hydrodynamic processes. In the long evolutionary and topological history of seagrass populations, ocean circulation has been a key driving force for the long‐distance dispersal of eelgrass, which greatly influences the biodiversity patterns of seagrass populations (Wu et al. 2018, Wu, Milne, et al. 2023). Therefore, reconstructing the main topological pathways of seagrasses in their distribution areas and their circulation‐driven mechanisms is of great theoretical and practical significance for understanding seagrass population dynamics and maintaining nearshore ecosystem stability.

To elucidate the mechanisms of target seagrass colonization, a multidisciplinary research approach can be applied to integrate evidence from various disciplines, such as population genetics and physical oceanography, to demonstrate the long‐distance dispersal pathways of seagrass reproductive branches or seeds. Based on a multidisciplinary research methodology, the study of the biological and physical mechanisms of current‐driven seagrass population proliferation will make the results more reliable. The results are expected to provide a multidisciplinary and integrated research paradigm for future exploration of the mechanism of long‐distance dispersal of seagrasses, provide solutions for the selection and delineation of protected areas and ecological restoration of seagrass beds in China, and provide an important scientific basis for the construction of a resilient network of marine protected areas.

## 2 Methods

### 2.1 Sampling

There exists a diverse array of seagrass species, with eelgrass being selected as the primary focus for discourse due to its ecological significance and widespread distribution in coastal marine environments (Olsen et al. 2016). Since eelgrass genome sequencing was completed in 2016 (Olsen et al. 2016), there has been a significant proliferation of eelgrass‐related molecular ecology studies, which have not only enhanced our understanding of its genetic makeup but also provided insights into its adaptive mechanisms in response to environmental changes (Wang et al. 2023; Pei et al. 2024; Yan et al. 2023a, 2023b). This positioning of eelgrass as an emerging model organism for studying seagrass evolution and genomics (Yu et al. 2023) is further supported by its role in critical ecosystem services such as carbon sequestration, habitat provision for marine fauna, and water quality improvement (Xu et al. 2022). These related developments and innovations of the above research bases and technological methodologies have enabled the successful localization of ecological circulation processes among different geographic populations of eelgrass. These studies employ various techniques including genomic analyses, population genetics, and remote sensing technologies that allow tracking genetic diversity across vast spatial scales and resolve the issue of their origins and expansion pathways, particularly in light of ongoing climate change impacts that threaten seagrass habitats globally.

Extensive sampling of the natural distribution of wild eelgrass populations in target marine areas is required. This approach needs to ensure that the different geographic populations of the existing eelgrass range from which the plants will be collected are covered. The selected populations exhibit both sexual and asexual reproduction capabilities. During the sampling, strategically designated sampling sites can be established to ensure comprehensive collection while minimizing the likelihood of resampling the same clone, with samples processed promptly. The specific methodology for the treatment of plant tissues is as follows: initially, the leaf sheaths are peeled off to reduce the epiphytes on the plants; subsequently, the basal meristematic tissue of the buds (5 cm) is selected as the target plant tissue, which is then thoroughly rinsed with phosphate‐buffered saline (PBS), transferred into a cryotube, and subjected to freezing in liquid nitrogen for 30 min prior to storage at −80°C (Olsen et al. 2016).

### 2.2 DNA Extraction, Whole Genome Resequencing, and Quality Checking

Using resequencing technology, DNA is extracted from leaf samples of seagrass to acquire genetic information pertaining to eelgrass populations within the study area. This process holds significant importance for understanding the ecological role of seagrass and its adaptive evolution. First, 100 mg of the tissue sample is collected that originates from healthy and vigorously growing individuals to guarantee the quality and representativeness of the extracted DNA. Then, the tissue samples are meticulously ground into fine powder, commonly employing the liquid nitrogen freezing grinding approach to avert DNA degradation during the grinding process. Next, the ground powder is transferred to a pre‐cooled centrifuge tube. The extraction of DNA is performed using the CTAB buffer under rigorous control over temperature and time. The CTAB method is a frequently employed technique for extracting plant genomic DNA, operating by disrupting cell membranes and precipitating nucleic acids to obtain highly pure DNA (Kamdem et al. 2023). The quality of the extracted DNA is evaluated using Qubit (Thermo Fisher Scientific, Waltham, MA, USA) and Nanodrop (Thermo Fisher Scientific, Waltham, MA), which are respectively utilized for quantitative analysis and spectrophotometric analysis, effectively detecting any potential contaminants or verifying whether the nucleic acid concentration meets the requirements for downstream experiments. Following successful execution of quality control processes, qualified DNA that adheres to the standards and shows no conspicuous signs of degradation or poses no risk of contamination is used for Illumina sequencing library construction. This step encompasses fragmentation, adapter ligation, and PCR amplification, and each stage demands precise control of reaction conditions to ensure the consistency and integrity of the library construction. The high‐throughput sequencing is performed using a NovaSeq 6000 sequencer, which is renowned for its capacity to provide rapid and efficient data generation, laying a solid foundation for subsequent data analysis (Modi et al. 2021). Through these meticulous steps, an abundance of information regarding the genetic variation of the targeted seagrass population can be obtained.

### 2.3 SNP Mapping, Calling, and Filtering

The raw data obtained from the Illumina platform are filtered using FASTP v.0.18.0 (Chen et al. 2018). FASTP functions as an efficient preprocessing tool for rapid sequencing data, automatically eliminating low‐quality reads and adapter sequences to enhance the accuracy and reliability of subsequent analyses (Chen et al. 2018). The software assesses each read segment according to predefined parameters and generates a detailed quality control report, thereby assisting in comprehending data quality (Chen et al. 2018). The filtered reads are subsequently aligned to the reference genome using a comparison software BWA v.0.7.12 with the mem algorithm (Li and Durbin 2009) and converted into the BAM format. BWA is widely utilized in genomic research; its mem algorithm is particularly proficient at aligning long reads while effectively mapping shorter segments onto the reference genome, which is essential for accurately determining individual sequence locations within samples, establishing a foundation for subsequent variant detection and functional annotation (Li and Durbin 2009). Upon completion of the alignment, the results are analyzed using the software picard (http://sourceforge.net/projects/picard/). Picard offers a suite of tools designed for BAM file processing that includes functionalities such as duplicate marking and coverage calculation. Duplicate reads are identified, and the depth and coverage of these marked reads are statistically assessed. Coverage statistics are calculated using software bedtools v.2.25.0 (Quinlan and Hall 2010), which provides a comprehensive set of command‐line utilities tailored for manipulating genomic intervals.

### 2.4 Genetic Diversity Analysis

Nucleotide diversity (π) for each population is calculated at all synonymous loci using VCFtools (https://vcftools.github.io/index.html), a widely utilized software suite specifically designed for the analysis of variant call format files, enabling one to efficiently manage large genomic datasets, which can provide insights into genetic variation within populations and highlights the potential impact of evolutionary processes and environmental factors on genetic diversity. Genomic heterozygosity is assessed within Python, which offers flexibility in data manipulation and statistical analysis through various libraries tailored for bioinformatics applications. The degree of linkage between loci is achieved based on genome‐wide single‐copy nuclear loci. Genetic penetrance is tested using the ABBA‐BABA statistic (also known as the D statistic), a method employed to detect introgression or incomplete lineage sorting among populations by comparing allele frequencies across different groups. Meanwhile, outgroups are used effectively to infer the ancestral status of genetic variation; they serve as reference points against which variations in other populations can be compared. Additionally, other genetic populations are used to determine the status of genetic variation among different populations to infer the degree of genetic differentiation, potential historical migration patterns, and demographic events that have shaped current population structures. These analyses ultimately aid in assessing conservation strategies by identifying genetically distinct lineages within eelgrass populations.

### 2.5 Population Structure

Based on the selected SNP data, initially calculate the genetic distances between samples. This process involves a thorough analysis of the SNP information for each sample to quantify genetic differences among them. By leveraging these genetic distances, subsequent clustering analyses are conducted to infer ancestral relationships and evolutionary histories across diverse populations. In constructing an evolutionary tree, the neighbor‐joining method (Vilella et al. 2008) is employed in conjunction with Treebest software, which effectively delineates systematic evolutionary relationships among samples and provides visual support for future research endeavors. Concurrently, to attain a more comprehensive understanding of population structure, PLINK (Chang et al. 2015) and GCTA (Yang et al. 2011) software are utilized to perform principal component analysis (PCA). This statistical approach facilitates the identification of primary sources of variation and elucidates potential patterns in population distribution. Moreover, an extensive analysis of population structure using Admixture software is executed (Alexander et al. 2009). This tool estimates individual proportions across various ancestral components, yielding critical insights into biological phenomena such as human migration events and adaptive processes (Alexander et al. 2009). Through the integration of these methodologies, not only can a quantitative assessment of genetic diversity be achieved but also a solid foundation for future related investigations be established.

### 2.6 Analysis of Reticulate Evolution

SplitsTree is used to assess the networked evolutionary processes in natural eelgrass populations (Huson and Bryant 2006). Initially, a custom Python script is utilized to generate a FASTA format file, wherein a random allele is selected to represent each individual locus, which can ensure dataset diversity and provide a robust foundation for subsequent analyses. Subsequently, randomly selected SNP sets are used to evaluate consistency across results, which are essential for understanding genetic variation and its adaptive expression under varying environmental conditions.

The NeighborNet method is adopted to construct a network illustrating species division patterns, which can effectively display complex data relationships and elucidate potential genetic connections and gene flow among species. Furthermore, phylogenetic trees are constructed using the maximum likelihood method, with topologies verified using IQ‐TREE v1.5.5 (http://www.iqtree.org/release/v1.5.5) to double‐check the obtained topologies. These findings illuminate the classification of genetic lineages and recent gene flow dynamics, offering new insights into how both internal and external factors influence the genetic structure of seagrass ecosystems. Additionally, these research outcomes may positively impact conservation biology, ecological restoration efforts, and related fields by identifying key genetic traits that can inform management strategies.

### 2.7 Temporal Calibration of Topological Events

Multiple Sequential Markov Coalescent (MSMC) method is performed for each genotype within the populations (Schifels and Durbin 2014; Schiffels and Wang 2020), which can effectively infer historical divergence times and evolutionary processes among different genotypes. A genome‐specific mask file is generated using bamCaller.py to ensure both accuracy and efficiency in data processing. Furthermore, variant call format (VCF) files containing all identified genetic variation information for each sample are generated using a custom Python script. Notably, due to the lack of direct fossil evidence in the genus Zostera, a divergence time of 9.86 ~ 12.67 mya is recommended. This divergence time is derived based on a clock rate of fourfold degeneracy reversal of homologous gene sequences resulting from a previously identified genome‐wide duplication event (dating back ~670,000 years). To gain deeper insights into the evolutionary history of the genus Zostera, multiple topological scenarios are constructed based on genetic structure results and screened for the optimal models of the topological history of eelgrass, which aim to reflect significant historical events.

### 2.8 Construction of Hydrodynamic Model in the Yellow Bohai Sea

The FVCOM numerical model not only accounts for various physical processes occurring in the ocean but also integrates meteorological factors and hydrographic characteristics to provide a more comprehensive representation of dynamic changes within the region. A three‐dimensional hydrodynamic model of wind‐tide‐thermostat in the target sea area is constructed (Changsheng et al. 2006), the multi‐layered circulation in the four seasons (spring, summer, fall, and winter) is simulated, and the computed data are compared with the floating data of the unit and the measured data of each tide gauge to verify the accuracy of the model. The calculated data are compared with the floating data of the unit and the measured data of the tide gauging stations to verify the accuracy of the model, and the circulation in the Yellow Bohai Sea is plotted schematically.

### 2.9 Lagrangian Water Quality Point Movement Simulation

The investigation into the reproduction period and seed drifting depth of seagrasses, in conjunction with surveys, is crucial for understanding the dynamic changes within marine ecosystems. By observing the growth and development of seagrass seeds across various temporal intervals, their optimal breeding periods can be delineated. Furthermore, comprehending the depths at which seeds float in water aids in assessing their survival rates and dispersal capabilities in natural environments. Combined with the survey to determine the time of eelgrass reproduction period and the depth of seed floating, the appropriate month and level were selected using the hydrodynamic model of the Lagrangian particle tracking module, which effectively simulates complex fluid dynamics while providing precise data support. In selecting appropriate release months and water depths, diverse climatic conditions, tidal fluctuations, and circulation patterns are taken into account to ensure that our chosen parameters accurately reflect real‐world scenarios. Upon determining specific release times and water depths, a corresponding number of particles are released into the simulated environment These particles represent seagrass seeds or reproductive branches that traverse defined paths driven by ocean currents, which can effectively simulate potential migration routes they may encounter under natural conditions, thereby enhancing our understanding of how seagrasses adapt to their surroundings and achieve self‐propagation. Finally, by generating trajectory maps of particle movement, which can not only visually illustrate the positional changes experienced by these particles over time but also analyze how different variables influence their movement pathways.

### 2.10 Localization and Validation of Eelgrass Natural Population Pathways

By integrating genetic studies with empirical survey results, a biological and physical validation of seagrass movement patterns is conducted, accurately identifying their settlement pathways. Analysis of the genomes from seagrass samples across various regions can reveal a significant correlation between genetic diversity and environmental factors and offer critical insights into the adaptive mechanisms of seagrasses within different ecosystems. Furthermore, during field investigations, water flow monitoring instruments to perform long‐term tracking observations of circulation patterns are employed to obtain more precise data, which can elucidate the physical mechanisms underlying seagrass dispersal through these currents, including how variables such as water temperature, salinity, and nutrient concentrations influence their distribution patterns. Concurrently, utilizing numerical simulation techniques can forecast the potential impacts of future climate change on these ecological processes. Ultimately, a map illustrating the settlement trajectories of seagrass species in the Yellow Sea and Bohai Sea regions is produced. This map not only depicts current species distributions but also indicates potential migration trends over time.

### 2.11 Assessment of Ecological Connectivity of Patches in the Eelgrass Colonization Path

In each ecological patch containing seagrass meadows, a systematic and comprehensive survey of biomass and species diversity is conducted. These surveys encompassed the growth status of seagrass itself and various aquatic organisms that coexist with or depend on seagrass beds, including fish, invertebrates, and other plant species. By collecting samples and performing laboratory analyses, the abundance and distribution characteristics of diverse organisms within these ecosystems under varying environmental conditions are accurately assessed. Furthermore, to deepen the understanding of the significance of water flow for ecosystem functionality, this study integrated simulations of water flow with comparative analyses examining how different water flow speeds and directions affect seagrass population size as well as surrounding ecological connectivity. Specifically, by developing numerical models, the impact of water flow on nutrient transport, light availability, and sediment movement across various scenarios is simulated; thus exploring how these factors collectively influence the development of seagrass beds and associated ecological communities. Ultimately, these results aim to elucidate the complex interplay between water flow dynamics and marine vegetation while providing a scientific foundation for future coastal ecosystem protection and management efforts.

## 3 Results and Discussion

**FIGURE 1:**
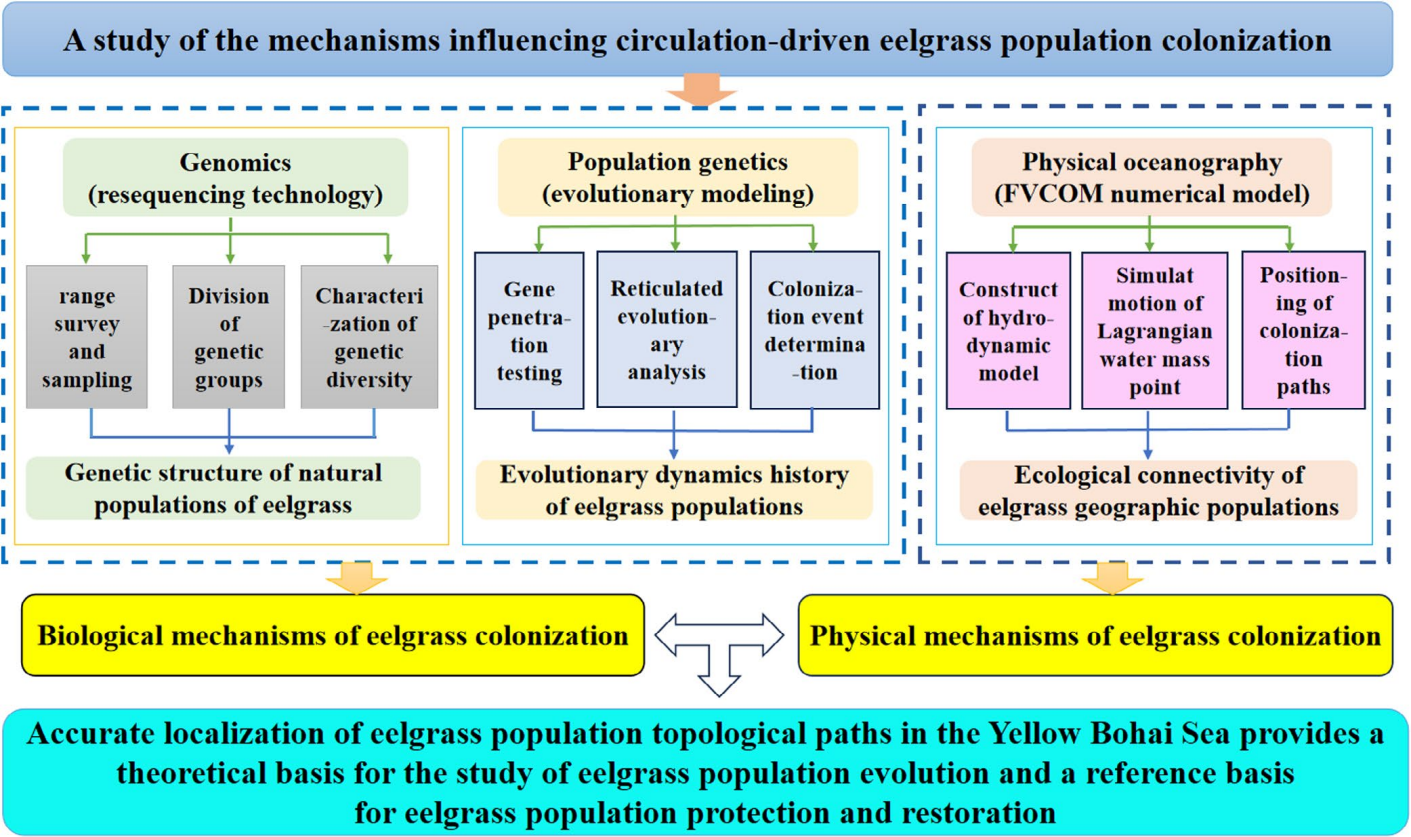
Technological route for the integration of various approaches to evaluate eelgrass topological pathways in the Yellow Bohai Sea.

Based on population genetics, the degree of genetic differentiation of natural eelgrass populations in the Yellow Bohai Sea can be clarified, and the population genetic structure is characterized. Based on the physical oceanographic method, the FVCOM numerical model is used to characterize the seasonal changes in surface circulation in the Yellow Bohai Sea and reveal the physical mechanism driving surface circulation movements affecting eelgrass population expansions. On the basis of the results of the current regional movement model, a more accurate eelgrass population expansion scenario is designed to reasonably deduce the dynamic history of eelgrass populations in China. By integrating genetic and physical mechanisms, the precise positioning of the eelgrass population expansion pathways in the Yellow Bohai Sea can be realized (Figure 1).

## 4 Conclusion

For angiosperms that live entirely in the ocean, seawater movement is a key factor that drives plant population reproduction and survival. Currently, most studies on seaweed population dynamics are based on biological theories and techniques, such as genomics, and there is very little research on the physical mechanisms of current‐driven population origin and migration. Owing to the lack of historical data on most seagrass populations, only some speculative suggestions can be proposed regarding the process of seagrass population expansion in the Yellow Bohai Sea, and there is a lack of theoretical verification and basic support at the physical oceanographic level. To determine the expansion process of seagrass populations in the nearshore waters of the target area, we conducted the large‐scale field sampling based on the natural distribution area of the target seagrasses can be conducted, the genetic diversity and differentiation process of seagrass populations using genomics and population genetics can be analyzed, and the history of their population dynamics can be speculated. Based on the FVCOM numerical model, seasonal changes in surface circulation patterns in the target area are clarified, and the physical driving mechanism of seagrass population genetic evolution by hydrodynamic processes is elucidated in conjunction with seagrass population dispersal time and distribution patterns.

## Author Contributions

**Zhaohua Wang:** conceptualization (equal), funding acquisition (equal), methodology (equal), writing – original draft (equal), writing – review and editing (equal). **Bin Zhou:** conceptualization (equal), methodology (equal), supervision (equal), writing – review and editing (equal). **Wenjie Yan:** conceptualization (equal), funding acquisition (equal), project administration (equal), writing – review and editing (equal).

## Conflicts of Interest

The authors declare no conflicts of interest.

## Data Availability

The authors have nothing to report.
